# Collectanea

**Published:** 1833-07

**Authors:** 


### COLLECTANEA.

### PATHOLOGY.

*On the Pathology of Purpura Hemorrhagica.* Extracted from a Paper by GEORGE GARDNER, Surgeon, Glasgow. [*Glasgow Med. Journal.*]

MR. GARDNER first gives a slight sketch of the various opinions which have been entertained by different pathologists respecting the disease in question; and then adduces the following cases, for the purpose of shewing that it is of an inflammatory nature, and that an antiphlogistic method of treatment is the only one from which any real advantage can be derived.

"In two cases, related by Dr. Parry, of Bath, both had decided symptoms of inflammation. The first was that of a lady, fifty years of age, of a fair complexion, and rather full habit, who for some days had laboured under slight febrile symptoms, with a full pulse, though but little thirst or fur on her tongue. Her skin was thickly covered with spots, small, and of irregular forms, not raised above the surface, of a dark logwood colour, and in no degree evanescent on pressure. Previous to Dr. Parry's first visit, she had been seen by an apothecary, who took ℥xiv. of blood from her arm. The blood was seen by Dr. Parry. Its surface consisted of a crust of coagulated lymph, as thick and tenacious as he ever witnessed in the most acute cases of rheumatism, pleurisy, or hepatitis. The cruor was also very firm and cohesive, and difficult of diffusion when shaken in the serum, and the proportion of crassamentum to that of the serum was very great. The patient expressed so much relief from the bleeding, that he thought himself justified in ordering it to be repeated. Relief was by this means obtained; and, under the use of some common refrigerants, she quickly recovered.

The second case was that of an officer whom he attended in conjunction with Mr. Atwood. The patient had been rather a free liver, and was suffering under considerable fever, apparently connected with inflammation of the testicle. The pulse being very hard and strong, the heat great, and the urine high coloured, Mr. Atwood was desirous of taking away blood, but was deterred by numerous petechiæ which appeared in the skin. Satisfied, after comparing this with the former case, that no hazard would arise from bloodletting, they agreed that the operation should be performed. The first bleeding afforded instant relief; and, after a second, the pulse was reduced to its natural state, the spots gradually disappeared, and the petechial disposition entirely ceased. The blood drawn had precisely the same appearance and tenacity as in the former instance.

"After relating these cases, Dr. Parry observes, that they strengthen an opinion which he had more than twenty years ago maintained, and which a large subsequent experience had tended to confirm; that in various diseases, among which may be reckoned inflammations, profluvia, hemorrhages, dropsies, exanthemata, and other cutaneous eruptions, and even the generality of nervous affections, there is one circumstance in common, which is an over distention of certain blood vessels, arising probably from their relative want of tone, or the due contraction of their muscular fibres.*

"A very striking case occurred to Dr. Bateman, at the Carey-street Dispensary, London. The patient was a feeble woman, about forty years of age. After fruitlessly taking bark and sulphuric acid for some time, she was slightly, but repeatedly, relieved by purgatives of calomel. After which her languor and weakness were diminished; but the petechiæ, which occurred principally on the extremities, were suddenly and finally removed, and her strength speedily restored, after a severe catamenial flooding. Dr. Bateman remarks, that this case, added to the two related by Dr. Parry, 'tend to throw a doubt upon our theories on the nature of petechial effusions.'†

"In a case which happened at the Edinburgh New Town Dispensary, the patient was a lad seventeen years of age; his diet had been meagre, and he had been accustomed to work in a cold and damp room. It was that form of the complaint which Dr. Willan calls purpura urticans. The eruption had the form of small hard purple coloured papulæ, intermixed with petechiæ; they occupied the ancles, knees, buttocks, and elbows. There were pains in these joints, accompanied with stiffness. An abscess which had formed on the parietes of the thorax, and had discharged itself three weeks before, had a dark purple colour, and was discharging a bloody sanies. When first seen he had much debility, and his countenance was extremely sallow, and expressive of languor. Pulse eighty-four, skin cool, tongue clean, and appetite tolerable. The petechiæ made their appearance suddenly, and were preceded by distinct rigor and febrile symptoms. The swelling and pain of the joints were abated by the use of purgatives; but, shortly after their operation, severe pain, with fulness and hardness of the abdomen, supervened, accompanied by vomiting and hiccough, full pulse, and foul tongue. These symptoms were relieved by bleeding to ℥xij.

* Ed. Med. and Surg. Journ., vol. v. p. 7. Many other cases, illustrative of the inflammatory nature of purpura, will be found in Dr. Parry's Posthumous Works, vol. i. They are there denominated inflammatory petechiæ.

† Edinburgh Med. and Surg. Journal, vol. vi. p. 124.

but recurred after two days in an increased degree, requiring three additional bleedings, each to ℥xiv., and the application of two blisters, before they were completely subdued. He bore the bleedings well. The coagulum was firm, and covered by a buffy coat of considerable thickness. In about a week after the first bleeding, the skin had regained its natural appearance, and the discharge from the abscess on the chest became healthy.*

"The peculiarities of this case appear to be, in the first place, the fact of the appearance of the petechiæ being ushered in by 'distinct rigor and febrile symptoms.' In the second place, the great quantity of blood taken for the reduction of the peritoneal inflammation, especially when the state of debility under which he previously laboured is taken into consideration. In the third place, the disappearance of the petechiæ shortly after the depletory plan had been put in execution, proving that the use of the lancet is as necessary for the dispersion of petechiæ in those who are labouring under great debility, as in those who are of a full and plethoric habit, and that nothing is to be feared in such cases from its use.

"On the 23d May, 1831, I was called upon to visit a young man, aged about seventeen, a silk weaver. He had a pale and exsanguineous appearance, though not much emaciated, and complained of much debility, accompanied with pain of head, lumbar region, and extremities. His neck, breast, and extremities, but particularly the upper, were studded with petechiæ of various sizes, but none larger than a split pea, some of a red, and others of a purple colour, each with a small white dot in its centre, and remaining unchanged by pressure. None of these spots were to be seen on the mucous membrane of either mouth, nostrils, or eyes. Pulse 104, full and bounding; respiration hurried, and accompanied with a slight cough; skin hot and dry; tongue pale red round edges, but covered in middle with an orange-coloured fur. The gums appeared to be ulcerated, and discharged blood on pressure; bowels open, but the stools were dark and fetid. The petechiæ had made their appearance about six months before I saw him, but up to that time was not prevented from following his usual occupation. He had, however, during that period, several attacks of hemorrhage from the left nostril, which always ceased spontaneously, without much loss of blood. For a few days previous to my visit, the petechiæ had been increasing in number, with an accompanying increase of debility. His diet had generally been of a nutritive quality, nor had he been too much excluded from out-of-door exercise.

"He was bled to ℥x; immediately after which he became sick, and vomited. A saline purgative was ordered to be taken as soon as the stomach became quiet. Next day I was informed that early that morning an epistaxis took place from left nostril, which was stopped by a neighbouring surgeon, by plugging, after the loss of several ounces of blood. The pain of head was gone; pulse ninety-

* Edinburgh Med. and Surg. Journal, vol. xii. p. 428.

six, and soft; and expressed himself as feeling better than he had done for some days. The blood taken from the arm coagulated without the separation of serum, and presented a pale red transparent jelly-like appearance, extending to the depth of a fourth of an inch, all under which was coal black. When the clot was broken down a sufficiency of serum was given out. He continued much in the same state, under the use of tonics and purgatives, till the 31st, when, in consultation with Dr. Hannay, he was found to complain of much headach; skin hot and dry; pulse 112, full and strong, and there was a continual oozing of blood from the left nostril, which was still plugged, and from the gums. There was much thirst and no appetite. From this condition it was judged advisable again to abstract blood. ℥xij. were accordingly taken with very considerable relief. The blood had altogether the same appearance as the former, with the exception of the coagulum being much firmer. Up to the third day from this time, there was great reason to believe that he was recovering. The appetite was considerably improved; the petechiæ were beginning to disappear, and the stools had nearly acquired their natural colour and consistence. These symptoms of improvement, however, speedily gave place to a state of much debility, which continued to increase, accompanied with a low muttering delirium for the two last days, and he died on the morning of 10th June. I regret that I had not an opportunity of examining the body.

"About a month afterwards I was requested to visit a sister of his, Mrs. G. aged about thirty, of a full and plethoric habit. I found her complaining of languor and lassitude, with slight pain in the lumbar region and thighs. On her breast and arms were scattered a number of petechiæ. They had existed for two days, and were very similar to those of her brother. Her gums were soft, but discharged no blood; nor had any hemorrhage taken place from the other passages of the body. Pulse ninety-six, very full. Tongue white. Bowels open. Blood was drawn to the amount of ten or eleven ounces; and she was ordered a dose of sulph. mag. In a few days she had completely recovered her strength, and the whole of the petechiæ were gone. The blood was cupped, and had the buffy coat.

"These cases, I apprehend, are sufficient to prove the inflammatory nature of purpura, although many more of a like character might be given."

Mr. Gardner concludes his very interesting paper by a few remarks tending to establish a strong resemblance, at least, between purpura and phlebitis; and he is of opinion that, although of late bleeding has been employed for the cure of the former disease, it has been practised empirically, rather than from correct pathological views. Of all the theories that have been advanced regarding the proximate cause of purpura, he affirms, and we perfectly agree with him, that none has done so much harm as that which ascribes it to general debility. Some nosologists have been greatly at fault in arranging purpura as a disease of the skin. Its place in the system of nosology is now, however, clearly indicated.

---

*Case of Partial Congestion of the Cerebrum; illustrative of the Plurality of the Organs and Faculties of the Intellect: with Remarks.* By William M. Fahnestock, m.d.

Much diversity of opinion still exists amongst philosophers in regard to the merits of the science of phrenology. At present it is attracting more attention than heretofore in this country, and there is every indication of a liberal disposition to give it that impartial consideration it so eminently demands from the investigators of truth. It has been neglected from prejudice, and the opposition of the metaphysicians, which have ever been arrayed against it; and especially from the reproach which has been so lavishly heaped upon craniology, a separate, and, in our opinion, a distinct science; as the absence of the evidences of the latter does not in the least impair the former. Phrenology, it must be confessed, is yet in a very imperfect state, though possessing high claims to favour, its fundamental propositions being founded in nature, and established, by well-attested facts, experiments and observations, on too firm a basis to be overthrown by ridicule, or the sophistry of schoolmen. Craniology, which is vulgarly deemed mere musings on an assemblage of bumps, but, by the scientific regarded as a classification of outward signs of the prominent development of the particular organs of the brain, depends on many contingencies, and must ever present imperfect indications. It asserts the possibility of recognising on the exterior of the cranium the seats of the various organs and faculties. But few contend that it is certain or infallible. Not so with phrenology or cerebral physiology. It includes the study of the structure of the encephalon, its functions, its relations, its derangements; it proposes to fathom the phenomena of the intellectual and affective faculties, to elucidate the philosophy of the mind. It is a subject susceptible of as clear demonstration as any of the other vital functions, and certainly much easier determined and understood.

Some metaphysicians who oppose in toto the idea of any connexion of the mind with physical organs, and regard the brain as a mere passive mass of nervous substance, when met by incontrovertible facts, declaim and protest most vehemently against drawing any deductions from a single case, or, in their own language, to erect a theory upon an isolated fact, while they, at the same time, build their whole system on hypothesis alone, without a single tangible fact to sustain their fairy web of fancy-work.

We do not intend by these remarks to enter into any discussion on the nature of the intellect, its essence, &c. but merely to introduce another pathological evidence, to the many already on record, showing that intelligence is connected with the cerebral organs; that those organs are multiple, and that the faculties are exercised according to the integrity of the structure of each individual organ, and are obstructed in part, or altogether, by congestions, or derangement in the various portions of the encephalon in which the different faculties are respectively located.

Baron LARREY, in his Surgical Memoirs, mentions several cases of wounds made by bayonets and swords penetrating the brain through the orbit of the eye, in which the memory for words was lost, but not of things. Dr. JACKSON has published a very interesting case of amnesia, in the Third Volume of this Journal, in which cerebral congestion suddenly induced suspended the memory for words, without any other disorder of the intellectual faculties; all the others being in full activity. Professor DICKSON, of Charleston, has also communicated a case,* in which there was a total loss of names, but a distinct memory of numbers, and power of rapid computation, remained. Plurality of organs and faculties would have given a ready solution to this case, without resorting to speculations on the problem of the mind having a greater aptitude to recognise the mathematical lines of figures. In the case which Professor WISTAR exhibited annually to the class in the University of Pennsylvania, if our recollection is distinct, there was a loss of ability to utter proper names when pressure was made on the anterior portion of the brain which was deprived of bone, the individual speaking with ease and fluency would stop articulating for a period sufficient to pronounce the name, and then go on to complete the sentence, as if unconscious of any interruption. Our reference to these few cases may suffice to screen us from the charge of laying too much might or stress on an isolated fact. An entire number of this journal would not contain the cases which might be adduced in confirmation of the plurality of the organs and faculties of the intellect.

The following case may afford additional argument to sustain the position.

In November last (1831,) John Flanagan, Esq., prothonotary of Franklin county, in this state, a gentleman of much intelligence and respectability, was attacked with influenza, then so prevalent throughout our country, which affected the upper forepart of his head especially; and particularly when he coughed, he suffered severe lancinating pain through the anterior lobes of the cerebrum, from the region of an inch and a half beneath the coronal suture to the inner angle of the eyes, which however did not prevent him from attending to his duties in court, then in session. One morning, after coughing severely, and experiencing much acute pain in the situation mentioned, which now continued without any cessation, he went into court, and in qualifying the jury found himself unable to name the parties in the suit, names with which he was perfectly familiar. He proceeded, "You do solemnly, sincerely and truly declare and affirm, that you will well and truly try the issue now

* American Journal of the Med. Sciences, vol. vii.

joined between—" and was interrupted by inability to articulate the names of the parties. After failing in two attempts, a gentleman of the bar rose in his place, and offered to discharge the duty, which Mr. Flanagan declined, and then proceeded the third time, but was arrested again as soon as he came to the names of the parties. He then turned to the judges, and told them that he was slightly indisposed, but would persevere; then placing his eyes on the names of the parties on the docket, both of whom were intimate personal friends, he proceeded the fourth time to the point at which he was before arrested, and was unable to proceed. The presiding judge named the parties, and Mr. Flanagan finished the affirmation without any further difficulty. The ability to articulate names was not only interrupted, but even the power to recall them was suspended. He could not recollect a single name one minute during the continuance of this congestion. The day following he was making an examination of the proceedings on the court record in a suit, and, in referring from the minute-book to the docket, he would forget entirely the names of the parties. He recurred three times to the original entry, and had at length to take the names on paper, and trace it on the other; for, as soon as he turned his eye from the name, it was entirely obliterated from memory. During this period his perception was clear, memory otherwise distinct, judgment sound, and business habits unimpaired. After taking a little revulsive medicine, in the course of a few days he returned to his duties, and has not experienced any further inconvenience.

We do not mean to deduce any theory from this case: we have no favorite theory to support on this subject. Our ambition is to elicit truth, and record facts; to be applied when the science of analogy shall come to reconcile the discrepancies of our present imperfect philosophy. Philosophy, like all abstruse sciences, is progressive in its advances, and its deductions are only to be received when supported by well-authenticated facts, or self-evident principles in strict analogy with nature. Our design is simply to add another fact to the mass of evidence, which illustrates and sustains the legitimate function of that delicate and wonderful organ, the encephalon.

One fact, we must remark, is worth a thousand conjectures; and we would be much more justifiable in proposing a theory founded upon a single fact, than to admit the vague assumptions and reveries of metaphysicians, which do not admit of being subjected to the test of demonstration. Instead of allowing the imagination to run riot in crude conjectures, and to revel in scholastic jargon, which has involved the science of physiology in so much obscurity, those who wish to arrive at any satisfactory conclusions respecting the manifestations of the intellect, the development of the mind, must examine minutely the structure of the brain, study attentively its functions, investigate its aberrations, and mark the abnormal condition of its diversified organs. An intimate acquaintance with the cerebral organization, cerebral physiology, and cerebral pathology, is indispensable to arrive at any certain knowledge of the operations of the intellect. Without these lights to direct us, we enter a labyrinth of doubt and darkness upon the very threshold of our speculations, and should continue to grope in fruitless research through bewildering mazes and interminable perplexities.—*American Journal of the Med. Sciences.*

---

### PRACTICAL MEDICINE.

*Treatment of the first Stage of Croup.* [From Mr. ADAMS' *Pathological Observations; Glasgow Med. Journal.*]

Dr. John D. Godman* narrates, that his children have been very liable to croup, and that he was informed by Dr. F. Vanderburgh of a very simple and efficacious method of arresting, at once, all the symptoms of this frequently fatal disease. Whenever children are threatened with an attack of cynanche trachealis, he directs a plaster, covered with dry Scotch snuff, to be applied directly across the top of the thorax, and retained there till all the symptoms disappear. Dr. V. stated, that he had found this remedy always effectual when applied in the first or second stages of the malady. Dr. Godman has tried this plaster repeatedly (made by greasing a piece of linen and covering it well with Scotch snuff), and has found it abundantly efficacious. His family has been saved a great degree of anxiety since, being quite prepared for the attacks, which are frequent in cold and wet seasons. Whenever a child is hoarse, or coughs with the ringing of croup, the plaster is applied, and, instead of watching all night, the family go to rest with a feeling of entire security.

Dr. Godman adds, that "in cases of croup the Scotch snuff (which I believe is prepared from tobacco-stems) is to be preferred." Dr. Pendleton, of New York, has tried other snuff without attaining his object, while with the Scotch the effect was certain. It has many advantages over tobacco-smoke, mentioned by Dr. Chapman. "It has never caused vomiting, vertigo, or any other distressing symptom in my experience," says Dr. Godman, "and this accords with the experience of both the physicians mentioned in this note."

Dr. G. informs us, by way of appendix, that Dr. V. is in the habit of deriving advantage from the external use of tobacco, in various cases, in which it is by no means generally employed, if its use be even thought of. To allay the irritative cough arising from different diseased states of the lungs, in diseases accompanied with chronic spasm, and in the reduction of hernia, by direct application of tobacco to the hernial tumor, &c., Dr. Vanderburgh has used tobacco externally with decided success.

* American Journal of the Med. Sciences, No. iv. Aug. 1828.

PHYSICAL SCIENCE.

*Cause of Crystallization.* M. CAUDIN has addressed an article on certain properties of atoms, the object of which is to point some particular cases of an extensive series of experiments regarding the immediate causes of crystallization. According to him, those bodies cleaving in a cube have for their primitive molecule a point or a right line; either an atom (as in metals); two dissimilar atoms (as in sulphuret of lead); three atoms, one of one kind, and two of another, (chloride of sodium): again, those molecules having for their form a double simple pyramid, or a prism of a number of sides, which is a multiple of four, may be assimilated to points when under different circumstances they form in groups around an accidental nucleus, and in this way they sometimes crystallize in cubes. Those bodies which cleave in a right prism, with any base whatever, have for their primitive molecule, either the polygon of the base, or a double simple pyramid, or a prism of a number of sides equal or double. When the primitive prism is not cleavable perpendicularly at the axis, it is because the molecules are polarized, that is, retained together either by an electric force, or by the force of affinity. The tetaedron, octaedron, and rhomboedron, have respectively for their primitive molecule, the first a square, or a rhomb; the second a double tetraedral or octaedral pyramid, simple or prismed, (prismèe), and the third a double hexaedral pyramid of the same nature. Finally, the absence of cleavage in these solids is owing to the want of continuity of the molecules. But in the tetraedron the want of continuity depends solely on this state of polarization, whilst in the octaedron and rhomboedron it results from the aggregation of the molecules.—*Dublin Journ. of Med. and Chem. Science.*

